# Effects of *Pseudomonas aeruginosa* on Microglial-Derived Extracellular Vesicle Biogenesis and Composition

**DOI:** 10.3390/pathogens8040297

**Published:** 2019-12-14

**Authors:** Leandra B. Jones, Sanjay Kumar, Courtnee’ R. Bell, Veolonda A. Peoples, Brennetta J. Crenshaw, Mamie T. Coats, Jessica A. Scoffield, Glenn C. Rowe, Brian Sims, Qiana L. Matthews

**Affiliations:** 1 Microbiology Program, Department of Biological Sciences, College of Science, Technology, Engineering and Mathematics, Alabama State University, Montgomery, AL 36104, USA; ljones@alasu.edu (L.B.J.); courtneerbell@yahoo.com (C.R.B.); vpeoples@alasu.edu (V.A.P.); bjcrenshaw0320@gmail.com (B.J.C.); mcoats@alasu.edu (M.T.C.); 2 Division of Neonatology, Departments of Pediatrics and Cell, Developmental and Integrative Biology, University of Alabama at Birmingham, Birmingham, AL 35294, USA; skumar@peds.uab.edu (S.K.); bsims@peds.uab.edu (B.S.); 3 Department of Biological Sciences, College of Science, Technology, Engineering and Mathematics, Alabama State University, Montgomery, AL 36104, USA; 4 Center for NanoBiotechnology Research (CNBR), Alabama State University, Montgomery, AL 36104, USA; 5 Department of Microbiology, School of Medicine, University of Alabama at Birmingham, Birmingham, AL 35294, USA; jscoff@uab.edu; 6 Division of Cardiovascular Diseases, Department of Medicine, University of Alabama at Birmingham, Birmingham, AL 35294, USA; glennrowe@uabmc.edu

**Keywords:** Extracellular vesicles, exosome, biogenesis, microglia, *Pseudomonas aeruginosa*

## Abstract

The packaging of molecular constituents inside extracellular vesicles (EVs) allows them to participate in intercellular communication and the transfer of biological molecules, however the role of EVs during bacterial infection is poorly understood. The goal of this study was to examine the effects of *Pseudomonas aeruginosa* (*P. aeruginosa)* infection on the biogenesis and composition of EVs derived from the mouse microglia cell line, BV-2. BV-2 cells were cultured in exosome-free media and infected with 0, 1.3 × 10^4^, or 2.6 × 10^4^ colony forming units per milliliter *P. aeruginosa* for 72 h. The results indicated that compared with the control group, BV-2 cell viability significantly decreased after *P. aeruginosa* infection and BV-2-derived EVs concentration decreased significantly in the *P. aeruginosa-*infected group. *P. aeruginosa* infection significantly decreased chemokine ligand 4 messenger RNA in BV-2-derived infected EVs, compared with the control group (*p* ≤ 0.05). This study also revealed that heat shock protein 70 (*p* ≤ 0.05) and heat shock protein 90β (*p* ≤ 0.001) levels of expression within EVs increased after *P. aeruginosa* infection. EV treatment with EVs derived from *P. aeruginosa* infection reduced cell viability of BV-2 cells. *P. aeruginosa* infection alters the expression of specific proteins and mRNA in EVs. Our study suggests that *P. aeruginosa* infection modulates EV biogenesis and composition, which may influence bacterial pathogenesis and infection.

## 1. Introduction

*Pseudomonas aeruginosa* (*P. aeruginosa)* is a Gram-negative, opportunistic pathogen that contributes to chronic airway infections in cystic fibrosis patients [1]. Moreover, *P. aeruginosa* infections have been implicated as the cause of life-threatening illnesses among immunocompromised individuals and burn victims who reside in healthcare facilities (e.g., hospitals, nursing homes [2], and rehabilitation centers [2]). According to the US Centers for Disease Control and Prevention, more than 6000 healthcare-associated multidrug-resistant *P. aeruginosa* infections occur annually; approximately 400 of these infections result in death. *P. aeruginosa* infection can spread systemically via a hematogenous infection. The bacterium can invade the central nervous system from the inner ear or paranasal sinus region. It can also be directly inoculated into the brain during head trauma, neurosurgery, or an invasive diagnostic procedure [3]. Because *P. aeruginosa* has become increasingly drug resistant, recent studies have dissected how *P. aeruginosa* disturbs immune cells and their ability to communicate with other cells using extracellular vesicles (EVs) [4,5,6,7].

EVs (30–1000 nm) are secreted from all cell types (e.g., T cells, mast cells, stem cells, microglia and endothelial cells) and are in many biological fluids (e.g., blood, saliva, breast milk, and urine) [7]. These “bioactive vesicles” facilitate intercellular communication, signaling, and immunoregulatory processes by passing molecular constituents between cells [7]. Molecular constituents, such as protein, miRNA, RNA, and lipids, function within EVs [8]. The presence of these functioning molecules makes EVs ideal for disease propagation. Several studies have examined EV biogenesis and composition and the roles of various agents during this process [9,10,11]. 

In this study, we report the effects of *P. aeruginosa* on the microglial cell line, BV-2, and the effects of *P. aeruginosa* on BV-2 EV biogenesis and composition. Microglial cells have an important role in the innate immune response in the brain via the release of cytokines after initial infection and cellular damage [12]. Further, microglial cells also initiate a pro-inflammatory response as a defense against toxic substances and pathogens. Cytokines (i.e., tumor necrosis factor alpha (TNFα), interleukin (IL) family) that are involved in the pro-inflammatory response are released within EVs [13]. This study examined the cytokine content packaged within microglia-derived EVs after *P. aeruginosa* infection; the findings further supported this phenomenon. We found that cell morphology (data now shown) [14], viability, and apoptotic markers were altered within 72 h after *P. aeruginosa* microglia infection. *Pseudomonas aeruginosa* infection also caused EV release and EV composition alterations. In summary, this study demonstrates that P. aeruginosa alters EV biogenesis and function, which may impact the outcomes of disease. 

## 2. Materials and Methods

### 2.1. Pseudomonas aeruginosa Strain

The *P. aeruginosa* laboratory strain PAO1 was generously gifted by Dr. Jessica Scoffield (University of Alabama at Birmingham) [15]. Pseudomonas isolation agar and Luria-Bertani broth were routinely used to culture PAO1 at 37 °C.

### 2.2. Cell Culture

Murine brain microglial BV-2 cells were given to us by Dr. Harald Neumann (University of Bonn LIFE and Brain Center, Bonn, Germany) [16]. The cells were cultured (cell passage number, 20–25) in Roswell Park Memorial Institute 1640 (RPMI) medium (Fisher Scientific) supplemented with 10% fetal bovine serum (FBS) and 1% penicillin streptomycin. The cells were maintained in a 5% CO_2_ atmosphere and were incubated at 37 °C to an approximately 70–80% confluency. 

### 2.3. Pseudomonas aeruginosa Infection on Microglial Cells

BV-2 cells (500,000) were seeded in T-25 flasks (Corning) and infected with 0 (control; no infection) and 2.6 × 10^4^ CFU/mL *P. aeruginosa* at 0.1 optical density (OD) in RPMI-1640 media with exosome-free FBS. Bacterial cells were prepared from an overnight culture and then subcultured to 0.1 OD. The bacterial pellet was obtained after centrifugation at 14,000 rpm for 10 min and was resuspended in RMPI-1640 medium. The culture medium was collected at 72 h (i.e., the experimental time point).

### 2.4. Cell Viability by Trypan Blue Exclusion Assay

BV-2 cells were examined for viability after control (no infection) or infection with 2.6 × 10^4^ CFU/mL *P. aeruginosa* (0.1 OD) at 72 h. All media was removed. The cells were gently scraped with a cell scraper and non-sterile 1× phosphate buffered saline (PBS) solution (1.5 mL). The solution was collected and gently mixed in 2.0-mL Eppendorf tubes. The cells were then counted, and viability was assessed using 0.4% trypan blue dye in a Countess™ Automated Cell Counter (Thermo Fisher Scientific). 

### 2.5. EV Isolation 

At various time points post-inoculation, groups of EVs were isolated from RPMI exosome-free cell culture media. After the media was collected, it was filtered through a 3-mL syringe with an attached 25-mm syringe filter (0.22-µm pore size). PBS (1×) was added to the media and centrifuged at 32,000 rpm for 70 min in a SW41Ti swinging bucket rotor at 4 °C using a Beckman Coulter Optima L-70K Ultracentrifuge. The supernatant was removed and approximately 500 µL resuspended EVs were collected from each sample. Following isolation, the EVs were quantitated using the Bradford-Lowry protein quantitation procedure (Bio-Rad Laboratories, Hercules, CA, USA) [17]. 

### 2.6. Quantification of EVs

EV size distribution and particle number per milliliter were quantified using NanoSight tracking analysis (NTA) (Nanosight NS300-LM10, Malvern Instruments, Inc., Malvern, UK) [18]. Briefly, the EV samples were diluted in PBS (1×) (1:1000) and placed inside a 0.3-mL disposable syringe. NTA visually analyzes and quantitates particle size by measuring Brownian motion as it relates to particle size in fluids. Five independent experiments were analyzed, and the results were calculated as mean ± standard deviation of the mean values. 

### 2.7. RNA Isolation from EVs

Total RNA was isolated from BV-2 cell-derived EVs using IsoMag Carbon-Based RNA Purification Magnetic Beads (Life Magnetics Inc., SKU LMCC250, Detroit, MI, USA). Prior to isolation, RNase was used to remove any residual RNA to avoid contamination. One day before infection, 5 × 10^5^ cells were plated in T-25 flasks. On the day of treatment, the medium was aspirated from each flask. Each flask was then rinsed with PBS and 6 mL exosome-free FBS RPMI-1640 media was added. The cells were treated with *P. aeruginosa*, and control wells with exosome-free FBS RPMI-1640 medium only were maintained. The media fractions were collected after infection treatment and the EVs were extracted as described in the EV isolation section. RNA was isolated from the exosomal fraction. Briefly, the EV fraction was lysed in 200 µL lysis buffer. The lysate was homogenized completely via vortexing to ensure that the solution was non-viscous. Seventy-five microliters of Life Magnetics beads were added directly to the exosomal lysate and were well-mixed using pipetting to achieve a uniform bead distribution. Neutralization buffer (125 µL) was then added to the suspension and it was thoroughly mixed using pipetting; 400 µL binding buffer was added during mixing. After thorough mixing, the lysate-bead solution was placed on the magnetic stand for 120 s and the clear supernatant was aspirated. The beads were washed using a two-step process; 750 µL and 500 µL wash buffer were used for the first and second washes, respectively. After the second wash, the mixture was transferred to a new 1.5-mL centrifuge tube, it was thoroughly mixed using pipetting, and placed on the magnetic stand for 30 s. The wash buffer was then removed completely and the beads were left to dry on the bench for 10–15 min at room temperature. After drying, 30 µL elution buffer was added to the beads, mixed, and incubated at 65 °C for 5 min, followed by centrifugation at 10,000× g for 2 min. The eluent was collected and quantified using a Nanodrop (NanoDrop 2000c, ThermoFisher Scientific, Waltham, MA, USA) spectrophotometer. 

### 2.8. cDNA Synthesis of Messenger RNAs

Total RNA (100 ng) isolated from the EVs was used to synthesize messenger RNA (mRNA)-specific cDNA using the mRNA cDNA synthesis kit, with poly (A) polymerase tailing (Applied Biological Materials (ABM), catalog no. G903), according to the manufacturer’s protocol. The cDNA was amplified using BrightGreen 5× qPCR master mix (ABM catalog no. MasterMix-5C). One microliter of each primer specific to mRNA CCL4 (TTCCTGCTGTTTCTCTTACACCT f, AAGGACGACAAAGAGAATGAGGA r 10 µM, IDT) and CCL5 (TTTGCCTACCTCTCCCTCG f, AAACGGATGGAGAGGGAGC 10 µM, IDT), along with the following control primers: 36B4 f (GGAGCCAGCGAGGCCACACTGCTG), 36B4 r (CTGGCCACGTTGCGGACACCCTCC), TBP f (ACCCTTCACCAATGACTCCTATG), TBP r (TGACTGCAGCAAATCGCTTGG), HPRT f (GTTAAGCAGTACAGCCCCAAA) and HPRT r (AGGGCATATCCAACAACAAACTT) were used in the amplifications. The quantitative polymerase chain reaction (qPCR) analysis was performed as follows: pre-incubation, 95 °C 10 min, followed by 40 cycles of 95 °C 10 s, 63 °C 15 s, and 72 °C for 5 s. The fold change in mRNA expression was calculated using the delta-delta CT (2-ΔΔCT) method. The CT values for the control and treated samples were normalized to the CT values of 36B4, TBP, and HPRT [19]. The fold change in mRNA expression for each treated sample compared with the control sample was determined. For qPCR, gene expression was calculated from four replicates for the respective treatments and from four independent experiments.

### 2.9. Detection of Proteins Via Dot Blot Analysis

Cell lysates and BV-2-derived EVs were evaluated using dot blot analysis. Protein quantitation was determined using the Bradford-Lowry protein quantitation procedure [9,20]. Briefly, EV proteins or cell lysates (0.8 μg) were lysed in 5× lysis buffer (Lane Marker Reducing Sample Buffer), boiled, and bound to nitrocellulose membranes for 10 min. Samples were blocked in Pierce Fast-Blocker with 0.09% Tween-20 for 5 min in a 60-mm diameter petri dish used for a reaction chamber. After blocking, the primary antibodies anti-sorting and assembly machinery component (samm50) (1:500 dilution, Thermo Scientific, catalog number (no.) PA5-56969), anti-cleaved caspase 3 (1:500 dilution, R&D system, catalog no. MAB835), anti-heat shock protein (anti-HSP) 90β (1:500 dilution, Thermo Scientific, catalog no. 37–9400), anti-HSP70 (1:500, Santa Cruz, catalog no. sc-32239), anti-lysosomal associated membrane protein (LAMP)-1 (1:250 dilution, Developmental Studies Hybridoma Bank (DSHB), catalog no. 1D4B), anti-IL-6 (1:500 dilution, DSHB, catalog no. CPTC-IL6-1), anti-IL-1β (1:500 dilution, Bioss antibodies, catalog no. bs-0812R-FR), and anti-tumor susceptibility gene (TSG) 101 (1:250 dilution, Thermo Fisher, catalog no. MA1-23296) were added to the plates and incubated for 1 h at room temperature. Following incubation, the membranes were washed three times with tris-buffered saline with 0.09% Tween-20 for 10 min. Next, each membrane was incubated with appropriate horseradish peroxidase-conjugated mouse (1:1000 dilution, Millipore Sigma, catalog no. AP124) or rabbit (1:1000 dilution, Thermo Scientific, catalog no. 31460) with secondary antibody for 1 h, and washed three times with TBS-T with 0.09% Tween-20 for 10 min. The nitrocellulose membranes were blotted using filter paper and developed using Super Signal West Femto Maximum Sensitivity Substrate (Thermo Scientific) for 2 min. The reactions were developed using a Bio-Rad ChemiDoc XRS+ System.

### 2.10. Evaluation of EV Proteins Via Enzyme-Linked Immunosorbent Assay

Enzyme-linked immunosorbent assay (ELISA) was performed to determine the proteins found in or on the EVs before and after *P. aeruginosa* infection. EVs (40 μg) were bound in 100 µL bicarbonate buffer (pH 9.5) in 96-well plates and incubated at 4 °C overnight. The following day, each plate was washed three times in 200 µL washing buffer (0.05% Tween-20 in PBS). The plate was then blocked using 100 µL blocking solution (5% non-fat dry milk and 0.05% Tween-20 in 1× PBS) for 1 h. After blocking, the primary antibodies anti-HSP70 (Santa Cruz, catalog no. sc-32239), anti-HSP 90β Thermo Scientific, catalog no. 37-9400), anti-cleaved caspase 3 (R & D, catalog no. MAB835), anti-LAMP-1 (DSHB, catalog no. 1D4B), anti-IL-6 (DSHB), anti-IL-1β (Bioss antibodies, catalog no. bs-0812R-FR), and anti-TSG101 (Thermo Fisher, catalog no. MA1-23296) were added to each sample with blocking buffer and incubated at room temperature for 2 h. After incubation, each plate was washed, horseradish peroxidase secondary antibody (Dako) was added, and the plate was incubated at room temperature for 30 min. The ELISAs were developed using SigmaFast *o*-phenylenediamine dihydrochloride peroxidase substrate (Sigma Aldrich) and read at a 405-nm wavelength using a Genemate UniRead 800 microplate reader. 

### 2.11. EV Transfer Assay 

BV-2 cells were plated at 500,000 cells per T175 flask. Following 24 h, each flask was washed with 1× PBS and filled with RMPI medium/exosome-free FBS. The BV-2 cells were then treated with BV-2-derived EVs from control cells and *P. aeruginosa-*infected cells at 10 µg and 40 µg [21]. At 72 h, the medium was discarded and cells were collected in 1× PBS. The solution was collected and gently mixed in a 50-mL centrifuge tube. The cells were then counted, and viability was assessed using 0.4% trypan blue dye in a Cell Countess Automated Cell Counter (Thermo Fisher Scientific). 

### 2.12. Statistical Analysis

The statistical analysis was performed using standard functions provided with Microsoft Excel Software (e.g., calculation of mean and standard deviation values). Excel was also used to perform the unpaired two-tailed *t* tests. For statistical comparisons between study groups for cell transfer anaylsis, two-way ANOVA was used followed by post Tukey testing. Data are displayed as mean ± standard deviation or standard error (as indicated). The probabilities of error were: (* indicated in figures) *p* ≤ 0.05, (**) *p* ≤ 0.01, (***) *p* ≤ 0.001, and (****) *p* ≤ 0.0001. 

## 3. Results

### 3.1. Pseudomonas aeruginosa Infection Reduces Microglial Cell Viability

To examine the effect of *P. aeruginosa* on the microglia cell line, BV-2, cells were infected with 0, 1.3 × 10^4^, or 2.6 × 10^4^ CFU/mL of *P. aeruginosa* for 24, 48, and 72 h in exosome-free media. Our objective was to determine the effect of *P. aeruginosa* on microglial-derived EVs. To determine the optimal infection concentration, we performed a series of dose and time-dependent response experiments [14]. The results indicated that a *P. aeruginosa* concentration of 2.6 × 10^4^ CFU/mL was an optimal sub-lethal infection concentration to determine cellular responses for the subsequent experiments.

**Figure 1:**
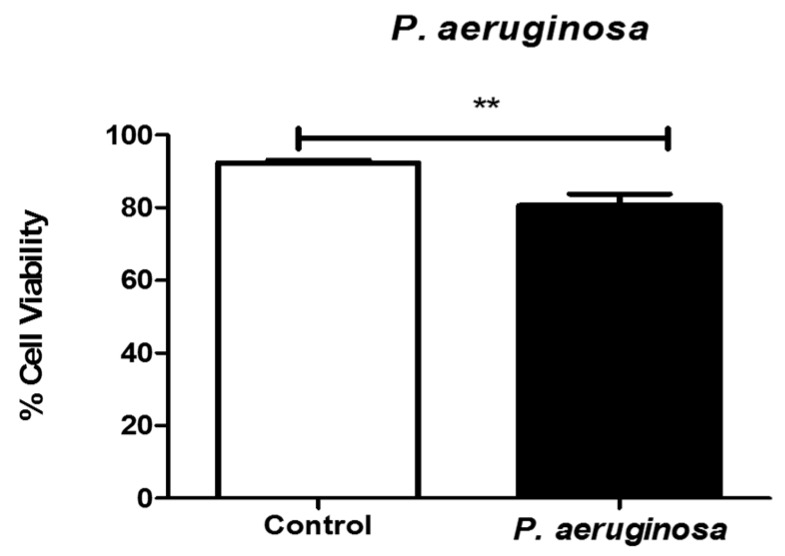
Viability of microglial cells after *Pseudomonas aeruginosa* infection. Cell viability was determined by trypan blue exclusion assay at 72 h post-inoculation. *P. aeruginosa* was inoculated at 2.6 × 10^4^ CFU/mL. Statistical significance is indicated as (**) *p* ≤ 0.01.

The trypan blue exclusion assay was performed to quantitate the numbers of viable cells present in BV-2 cell suspensions after *P. aeruginosa* infection. At 72 h, with 2.6 × 10^4^ CFU/mL infection, cell viability was significantly decreased by approximately 12% (*p* ≤ 0.01), compared with the control cells (Figure 1). Our results indicated that by 72 h, *P. aeruginosa* infection significantly reduced the viability of microglial, BV-2 cells.

### 3.2. Pseudomonas aeruginosa Infection Reduces BV-2-Derived EVs

**Figure 2:**
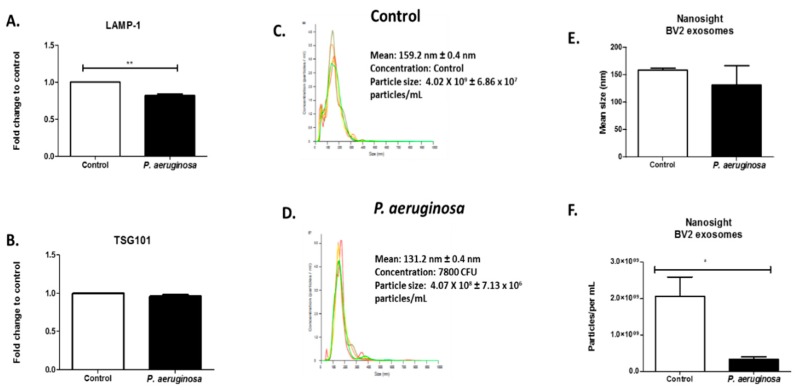
Quantitation of BV-2-derived EVs after *Pseudomonas aeruginosa* infection. ELISAs of BV-2-derived EVs at 40 μg were tested for the presence of LAMP-1 (**A**) and TSG101 **(B**) proteins. Histogram plot of the concentration of control EVs (**C**) and *P. aeruginosa*-derived EVs, 2.6 × 10^4^ CFU/mL initial concentration (**D**) (N = 1). Different colors indicate different groups of EVs. Size determination and quantitation of BV-2-derived EVs using NTA (**C**–**F**). Statistical significance is indicated as (*) *p* ≤ 0.05 and (**) *p* ≤ 0.01.

To determine the effect of *P. aeruginosa* on BV-2-derived EVs, BV-2 cells were cultured in exosome-free media and infected with *P. aeruginosa* (2.6 × 10^4^ CFU/mL) for 72 h. The EVs released into the cultured media were isolated using a series of high-speed ultracentrifugation steps [9] and were then characterized. EV presence was also confirmed with ELISA using EV markers, and TSG101 and LAMP-1 proteins (Figure 2A,B). LAMP-1 protein is a model EV marker. It is a marker for late endosomes and was used to confirm that the EVs completed endocytosis and were transported to endosomal organelles [22,23]. We found a significant decrease in the presence of LAMP-1 in the EVs derived after infection, compared with the control (Figure 2A). EV marker, TSG101, was also present within both groups of BV-2-derived EVs (Figure 2B). 

EV size was determined using NTA (Figure 2C–E). Illustrative histograms of control-derived EVs and EVs derived at 72 h infection revealed that control BV-2-derived EVs had a mean concentration of 4.02 × 10^9^ ± 6.86 × 10^7^ particles/mL. *Pseudomonas aeruginosa-*derived EVs had a mean concentration of 4.07 × 10^8^ ± 7.13 × 10^6^ particles/mL (Figure 2C,D). The NTA revealed strong peaks at the mean EV size at 159 ± 0.4 nm in the control microglial-derived EVs and 131.1 ± 0.4 nm for *P. aeruginosa* microglial-derived EVs (Figure 2C,D). This result indicated there was a decrease in EV size when infection was present. Infection with *P. aeruginosa* significantly (*p* ≤ 0.05) decreased the EV particle number by 1.77 × 10^7^ particles/mL (Figure 2F). 

### 3.3. Cytokine Response to Pseudomonas aeruginosa Infection in EVs

**Figure 3:**
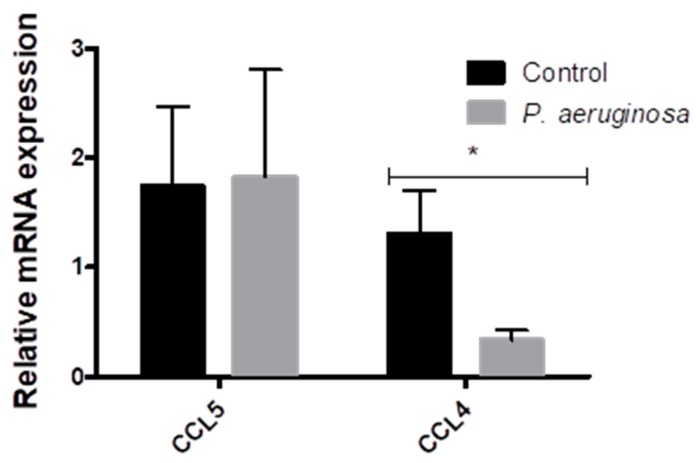
Changes in CCL5 and CCL4 expression in BV-2 cell-derived EVs. CCL5 and CCL4 expression in EVs derived from BV-2 cells at 72 h post-inoculation with *P. aeruginosa*. CCL5 and CCL4 mRNA expression normalized to 36B4, TBP, and HPRT, relative to the control. The results are presented as mean ± SEM values. Statistical significance is indicated as (*) *p* ≤ 0.05.

Small RNAs participate in disease initiation and progression [24,25]. EVs carry a variety of mRNAs, such as the chemokine ligands. We further evaluated the effects of *P. aeruginosa* infection on the release of CCL4 and CCL5 mRNAs into BV-2 cell-derived EVs (Figure 3). BV-2 cell-derived EVs were collected at 72 h post-inoculation with *P. aeruginosa* and evaluated for the presence of CCL4 and CCL5 mRNA. *Pseudomonas aeruginosa* infection elicited no change in the presence of CCL5 mRNA in EVs, compared with the control (Figure 3). CCL4, showed a significant decrease (*p* ≤ 0.05) in expression post-inoculation in BV-2 cell-derived EVs when compared to the control EVs.

### 3.4. Pseudomonas aeruginosa Induces Cellular Stress Response 

**Figure 4:**
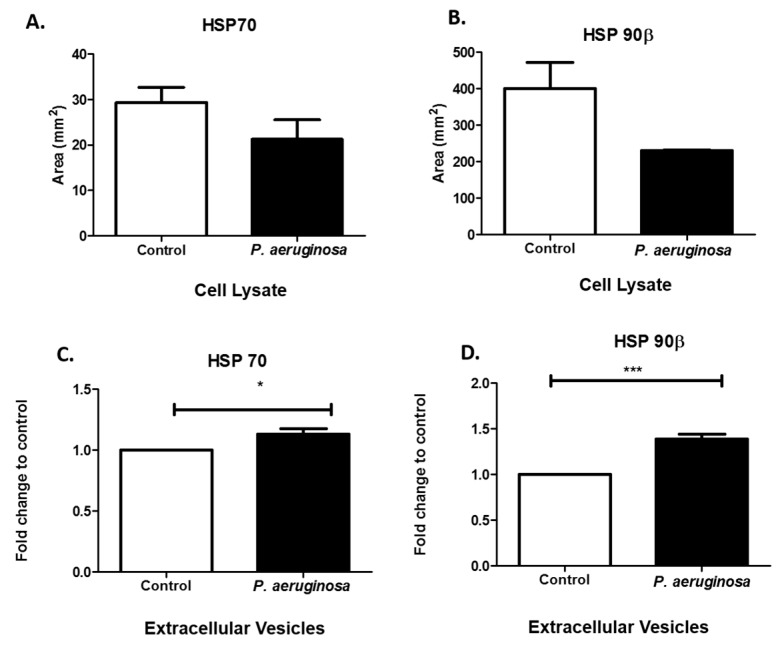
Stress-induced protein expression within infected BV-2 cell lysates and BV-2-derived EVs. HSP70 and HSP90β were found in BV-2 lysates after 72 h *P. aeruginosa* infection using dot blot analysis (**A**,**B**). HSP70 (**C**) and HSP90β (**D**) were detected in EVs at 72 h post-inoculation and confirmed using ELISA. Experiments were performed using four or five experiments. Statistical significance is indicated as (*) *p* ≤ 0.05 and (***) *p* ≤ 0.001.

As molecular chaperone proteins, HSPs facilitate the synthesis and folding of proteins. HSPs can be expressed in response to stressful environments. These chaperone proteins are involved in the direct maintenance of protein structure and control apoptotic pathways [26]. Mammalian cells express high levels of stress proteins after exposure to bacteria or bacterial proteins [26]. To determine how BV-2 cells responded to the stress of *P. aeruginosa* infection, we evaluated HSP70 and HSP90β in BV-2 cell lysates. Both HSP70 and HSP90β were downregulated in response to *P. aeruginosa* infection (Figure 4A,B). We found that both HSP70 (*p* ≤ 0.05), anti-death and immunoregulatory pro-inflammatory protein, and HSP90β (*p* ≤ 0.001), immunomodulator and pro-immune responsive, were both significantly upregulated in expression in the infection groups compared with the control groups (Figure 4C,D), in EVs. The results indicated that *P. aeruginosa* greatly affected the expression of stress-related proteins inside the cell and within the released EVs.

### 3.5. Expression of Immune Regulators are Present in EVs

**Figure 5:**
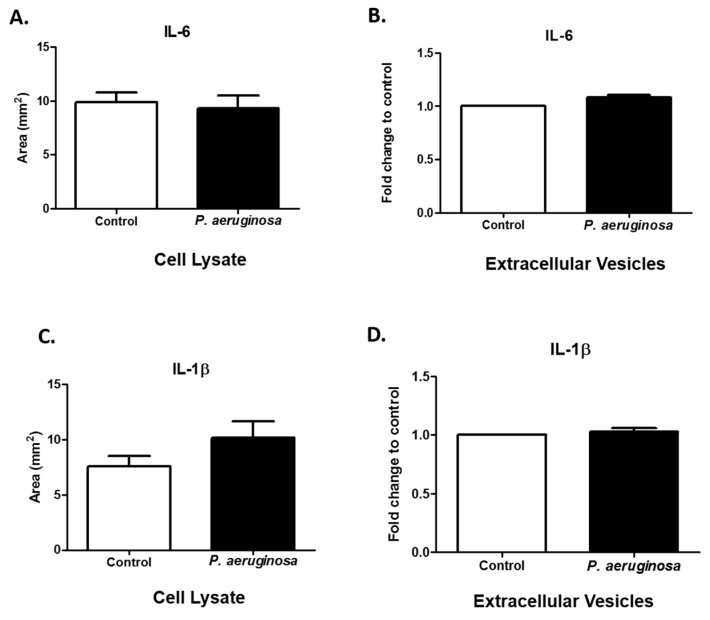
Expression of immune regulators. IL-6 (**A**) and IL-1β (**C**) were detected in cell lysates (control or *P. aeruginosa*-infected (2.6 × 10^4^ CFU/mL)). IL-6 (**B**) and IL-1β (**D**) were detected in EVs (control or *P. aeruginosa*-infected (2.6 × 10^4^ CFU/mL)). Experiments were performed using four or five independent experiments. The results are presented as mean ± SEM values.

Interleukins are pro-inflammatory cytokines that are vital for cellular signaling and inflammatory feedbacks [27]. The immune system depends essentially on interleukins. IL-6 acts as a first line of defense system for many cell types (e.g., stromal [28], epithelial [28], hematopoietic [28], and microglial cells) [29] by releasing signals to nearby and distant cells. Gram-negative bacteria stimulate IL-6 production in microglial cells [30]. IL-6 release enhances survival rates of infected cells [30]. Here, we found IL-6 in BV-2 control cell lysate and *P. aeruginosa-*infected cell lysate (Figure 5A). We also found IL-6 in both groups of EVs (Figure 5B). A representative dot blot image of IL-6 expression in cell lysate can be found in Supplementary Figure S1.

The pro-inflammatory cytokine, IL-1β, has critical roles in the activation of many inflammatory pathways and adaptive immunity. IL-1β levels can modulate in response to bacterial infection [31]. IL-1β levels in cell lysates were slightly increased after *P. aeruginosa* infection, compared with the control group (Figure 5C). The results of another study indicated there is a statistically significant difference in intensity of expression (*p* ≤ 0.001) of IL-1β in control compared with *P. aeruginosa* groups of cell lysates [14]. In this study, we found IL-1β within the packaging of the control and *P. aeruginosa-*infected BV-2-derived EVs. Taken together, these results suggest that EVs act as cargo carriers for cytokines produced by BV-2 cells (Figure 5D).

### 3.6. Pseudomonas Aeruginosa Decreases Samm50 Expression in Cell Lysates 

**Figure 6:**
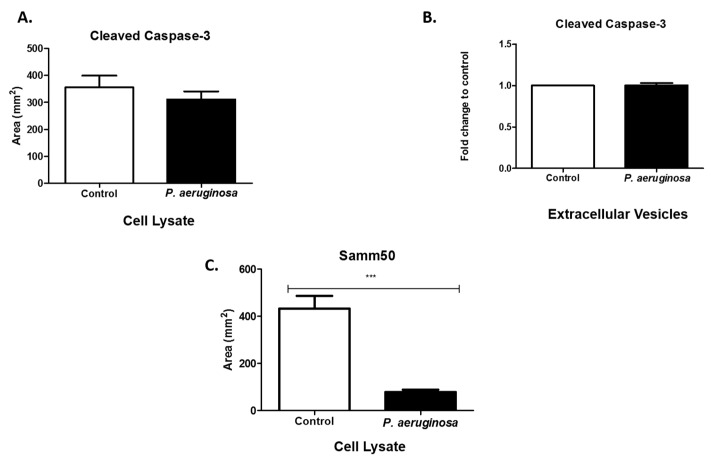
Expression of apoptotic proteins. Cleaved caspase-3 (**A**,**B**) were expressed in cell lysates and EVs. At 72-h post-inoculation, protein was detected via dot blot analysis and ELISA. Samm50 (**C**) presence was observed in cell lysates using dot blot analysis. Experiments were performed using four or five independent replicates. Statistical significance is indicated by (***) *p* ≤ 0.001.

Caspases are modulators of cell death in response to stimuli (e.g., infections, stress) [32]. We evaluated cleaved caspase-3 expression in cell lysates and EVs. The presence of cleaved caspase-3 indicates the manifestation of apoptotic pathways within the cell as it responds to the bacterial infection [33]. Cleaved caspase-3 was expressed in cell lysates and slightly decreased as a result of *P. aeruginosa* infection (Figure 6A). However, in EVs there was relatively no change between the control and the *P. aeruginosa-*infected groups (Figure 6B). Samm50 is a component of the sorting and assembly machinery complex (SAM). It is in the outer membranes of mitochondria and has an essential role in the maintenance of mitochondrial cristae structure [34,35]. Samm50 targets contaminants and bacterial effector proteins that affect host mitochondria. We found that *P. aeruginosa* significantly decreased Samm50 (*p* ≤ 0.001) expression, compared with uninfected cells (Figure 6C). Previous studies have found that Samm50 depletion protects cells from caspase-3-induced cell death [34].

### 3.7. EV Treatment Decreases Cell Viability

**Figure 7:**
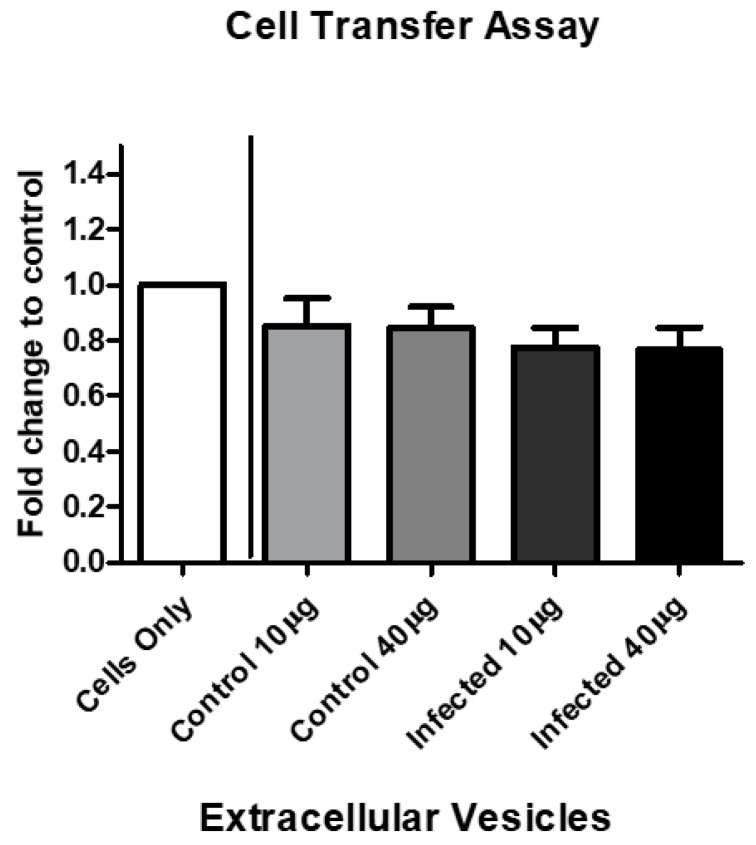
BV-2-derived EV treatment decreases cell viability. BV-2 cells in exosome-free medium were treated with BV-2-derived and *P. aeruginosa*-derived EVs at 10 µg/mL or 40 µg/mL for 72 h. Cell viability was assessed by trypan blue exclusion assay. The mean values for five independent experiments are presented as fold changes. The results are presented as mean ± SEM values.

The viability of BV-2 cells after EV treatment decreased by 72 h. We speculated that the cell viability decreases observed in Figure 1 could partially be mediated by an unknown EV-associated mechanism. We observed the effects on the BV-2 cells after EV treatment with BV-2-derived EVs (control) and EVs derived from *P. aeruginosa* infection (infected). By 72 h, infected BV-2-derived EVs decreased BV-2 cell viability at both concentrations (10 µg and 40 µg) by approximately 0.23–0.24 fold, compared with the control cells (Figure 7).

## 4. Discussion

Gram-negative bacteria are associated with approximately two million nosocomial infections and 90,000 fatalities annually in the USA [36]. Due to high rates of *P. aeruginosa* infection, the increases in multi-drug resistant *P. aeruginosa*, and the evolution of the bacterium against antibiotics (e.g., cephalosporins, carbapenems, and aminoglycosides), it is responsible for fatal health-related threats worldwide [37]. In the USA, the number of infections is increasing and the number of effective treatments is decreasing. Thus, new biological information regarding pathogenesis is needed to develop effective treatments against pathogenic microbes. 

In our study, we performed an evaluation of EV biogenesis and composition to understand the effect of *P. aeruginosa* on the microglia cell line, BV-2. Microglia are the resident brain macrophages that maintain tissue homeostasis. Microglia function is one of the primary defenses against bacterial infection and brain injury, and stimulate tissue repair [38]. Microglia recognize many Gram-negative pathogenic bacteria that can colonize the central nervous system [39]. In addition to *P. aeruginosa*, *Escherichia coli* and *Vibrio vulnificus* have effects on microglial cells [40,41,42]. Hoogland et al. developed a live *E. coli* mouse model that mimics the delirium-associated infection [41]. They found that compared with control mice, mice infected with live *E. coli* have microglial activation by 72 h post-inoculation. Using flow cytometry analysis, they also found that by 72 h, microglia from *E. coli*-infected mice had increases in mean cell size and CD45 expression, compared with the controls [41]. Mayer et al.’s results suggested that *V. vulnificus* MO6-24/O lipopolysaccharide activates rat microglia in vitro and stimulates the release of superoxide anion in vivo [42]. Other species of Gram-negative bacteria likely enter and replicate in the central nervous system [43]. However, few to no study results have been published on this topic. 

Disease-associated proteins (e.g., β-amyloid [44], superoxide dismutase [45], and α-synuclein [46]) are released from cells during the EV response. The secreted proteins likely participate in the pathogenic response via interaction with recipient cells [47]. Alpha-synuclein can be found in the plasma and cerebrospinal fluid of humans, which further supports the hypothesis that EVs can be used as biomarkers for disease [47]. The results of infection biology studies indicate that EVs are released by mammalian cells infected with bacteria, infectious prion proteins, and viruses [48]. 

Using an in vitro model for human physiology, we observed the release of EVs in BV-2 cell culture media in response to *P. aeruginosa* infection. *Pseudomonas aeruginosa* cannot be isolated from the body to distinguish which EVs were produced by which organism. Using NTA, we found a significant decrease in EV particle number/mL after *P. aeruginosa* infection. Decreased EV numbers correlated to a significant decrease in cell viability after *P. aeruginosa* (2.6 × 10^4^ CFU/mL) infection (Figure 2). At 72 h post-inoculation, the EV particle/cell number was 706 EVs/per control cell versus 205 EVs/per infected cell. This result indicated that in response to infection, EVs were being released at slower rates, which correlated with the decreasing cell survival rate. When used in combination, the qPCR, dot blot analysis, and ELISA allowed us to examine mRNA, EV markers (e.g., LAMP-1, TSG101), and proteins (e.g., HSPs, ILs, caspases, etc.) that are found internally and externally on EVs (Figure 3, Figure 4, Figure 5 and Figure 6).

EVs have a vital role in cell-to-cell communication by transferring mRNA through an endocytic process. While we tested miRNAs and mRNA for expression within BV-2-derived EVs (Supplementary Figure S3), the most significant findings were those related to the chemokine ligand family. The chemokine ligands, CCL4 and CCL5, have active roles in the inflammatory response in tissues. Our results indicated that chemokine ligands were in BV-2-derived EVs; specifically, *P. aeruginosa* infection caused a significant decrease in CCL4 packaged within EVs (Figure 3). We found that these two chemokines were produced in BV-2-derived EVs. To our knowledge, this finding is the first in the context of *P. aeruginosa,* chemokine ligands, and EVs. The chemoattractant, CCL4, is a major chemokine for lymphocytes, naïve dendritic cells, monocytes, and natural killer cells [49]. The downregulation of CCL4 production by microglial-derived EVs in response to *P. aeruginosa* suggested that microglial cells do not have an initial role in immune response activation. These findings were not consistent with the findings of Sun et al., which suggest that *P. aeruginosa* infection causes an increase in CCL4 production and subsequently initiates the host immune response [49]. The inconsistent findings could be due impartially to Sun et al. using mast cells rather than microglial cells in their study. 

HSP70 is packaged within EVs released from astrocytes under stressful conditions, such as heat or oxidative stresses and subsequently has a pro-survival effect on neurons [50]. HSP70′s protection of brain cells against other stressors could be due to how it functions during the prevention of damaging pro-inflammatory responses [51]. Our results indicated that HSP70 was significantly expressed in BV-2-derived EVs at the 72-h time point. Therefore, it likely has a critical role in cytoprotection (Figure 4C). Toll-like receptors (TLRs) use HSP70 as a ligand on immune cells (i.e., microglia) [52]. In our previous study, we did not find TLR2 [14], but that does not exclude participation of other TLRs. Other TLRs would need to be tested to determine which TLR contributes to this pathway. HSP secretion also occurs independently, or in the lack of cell death, which suggests that secretion could be occurring via active mechanisms such as EVs. 

HSP90β levels of expression significantly increased within the BV-2 EVs derived after *P. aeruginosa* infection (Figure 4D). The effects of HSP90β inhibition in the brain are not well-studied. However, HSP90β does regulate HSP70 induction [53]. This study found that *P. aeruginosa* also generally released EVs. However, we hypothesize that the predominant EV populations are derived from the BV-2 cell line. Our results suggested that both HSP70 and 90β are upregulated in BV-2-derived-EVs in the presence of *P. aeruginosa* infection. We also found HSP70 and HSP90β in cell lysates. HSPs protect against an array of stress-related factors, including bacterial infection, hyperthermia, oxygen radicals, heavy metals, and ethanol [20]. 

Pathogens also use the host’s HSPs to their specific advantage. Henderson et al. found that bacteria express numerous molecular chaperones on their cell surfaces. They can secrete these into the extracellular environment to act as virulence signals [54]. Hence, HSP70 is connected to microbial pathogenesis, immune responses, and apoptosis [7]. The stress response is vital for bacteria during adaptation to fluctuations in their biological state. HSP70 proteins and their cooperating chaperones create complex systems of protein-folding machines in prokaryotes and in eukaryotic cellular compartments and are expressed during stress conditions. Molecular chaperones regulate many tactics used by bacterial pathogens, including entry into the host, and replication and survival after entry [55]. A recent methodology to counteract bacterial infections is to exploit HSP70 and its co-chaperones as possible therapeutic targets. This approach allows prokaryotes to proceed through stress-induced changes caused by host responses or antibiotics [56]. Therapeutic treatment or a vaccine vehicle targeting these proteins may be an effective approach to limit bacterial pathogenesis.

To evaluate the role of immune regulators and *P. aeruginosa* infection, we examined IL-6 and IL-1β in BV-2 *P. aeruginosa*-derived EVs and cell lysates. Interleukins have a key part in systemic immune protection against infections, particularly bacterial infections [57]. Interleukins protect by activating the adaptive immune response that occurs after apoptotic reactions. The multifunctional cytokine, IL-6, has a central role in host immunity and it was present in EVs and cell lysates of both BV-2 groups, the control and *P. aeruginosa-*infected groups. IL-1β is a potent stimulator of IL-6 production in central nervous system astrocytes [58]. Microglial cells release IL-6 and IL-1β, which are both upregulated [29]. Our study found that interleukins were shuttled into the endocytic EVs from the cell in response to the Gram-negative pathogen, *P. aeruginosa*. We also tested and found immunomodulatory agents (IL-6 and IL-1β) and inflammatory response provoker, TNFα, in BV2-derived EVs. This result further supports the presence of immune regulators as part of the contents of EVs, in response to infection [14]. TNFα participates in biological effects including cell growth, cell differentiation, and cytotoxicity and, most importantly, in our study, anti-infection [59,60]. Finally, in a previous study, when we compared cell lysates of control to *P. aeruginosa*-infected cells, we found a statistically significant increase in intensity of *P. aeruginosa*-infected cells during evaluation of NLR family pyrin domain containing 3 (NLRP3) and B-cell lymphoma 2 (BCL-2) [14]. A list of proteins that we tested for presence in the BV-2-derived EVs is presented in Supplementary Figure S2. 

Fundamentally, EV functionality and characteristics (e.g., cell-to-cell communication) suggest that they have one or more roles in the pathogenesis of bacterial infections, whether it is host protection or assisting during bacterial invasion, or both. Here, we found that BV-2 viable cell number decreased after treatment with EVs derived after *P. aeruginosa* infection (Figure 7). This finding directly correlated with the cell viability results (Figure 1). Although *P. aeruginosa* infection causes cell viability to decrease over time, our results (Figure 7) suggested that in the presence of EVs, viable cells were also affected by molecular constituents packaged in the EVs. This area of EV research is not well-understood and requires further study. 

## 5. Conclusions

Bacteria, particularly Gram-negative bacteria, can release EVs for intercellular communication in prokaryotic and eukaryotic cells [7]. Intercellular communication can be mediated through direct cell-to-cell communication or transfer of secreted molecular constituents [47]. During bacterial infections, EVs can have different roles during the pathogen’s life cycle. EVs activate immune responses that combat the bacterium or assist the bacterium in the spread of the infection by spreading its virulence factors, or both [7]. Our findings suggested that EVs have an important role in infection biology. These vesicles may be directly pathogen-derived or released from microglial cells. In both cases, understanding their complex roles in cell-to-cell communication and immune modulation is important. A comprehensive investigation of EVs derived from non-infected and infected hosts is still needed to explain mechanisms related to pathogenesis and innovative therapeutics. These findings suggest that the potential to use EVs as a biomarker may guide the advancement of novel therapeutics for challenging and defiant pathogens. Furthermore, these studies shed light on the impact of Gram-negative bacteria on the central nervous system and EV biogenesis. In future studies, we will compare the effects of Gram-negative bacteria on EV biogenesis in vivo.

## Supplementary Materials

The following are available online at https://www.mdpi.com/2076-0817/8/4/297/s1, Figure S1: Dot blot analysis of cell lysates. Representative dot blot analysis of BV-2-derived cell lysates at 0.8 μg were tested with IL-6. The quantitative results can be found in Figure 5A. Figure S2: Additional proteins tested for expression in BV-2-derived exosomes and cell lysates. Figure S3: Additional miRNA and mRNA tested for presence in BV-2- derived exosomes and cell lysates.

## Data Accessibility

The original data will be maintained by the corresponding author. Information pertaining to the data sets will be made available upon written request.

## References

[1] Lim S.M., Webb S.A.R. (2005). Nosocomial bacterial infections in Intensive Care Units. I: Organisms and mechanisms of antibiotic resistance. Anaesthesia.

[2] Santus G., Brun C., Viani P., Pirali F. (2005). Nosocomial infections in the rehabilitation department. Eur. Med..

[3] Mesaros N., Nordmann P., Plesiat P., Roussel-Delvallez M., Van Eldere J., Glupczynski Y., Van Laethem Y., Jacobs F., Lebecque P., Malfroot A. (2007). Pseudomonas aeruginosa: Resistance and therapeutic options at the turn of the new millennium. Clin. Microbiol. Infect..

[4] De Toro J., Herschlik L., Waldner C., Mongini C. (2015). Emerging roles of exosomes in normal and pathological conditions: New insights for diagnosis and therapeutic applications. Front. Immunol..

[5] Wang J. (2017). Novel Implications of Exosomes in Diagnosis and Treatment of Cancer and Infectious Diseases.

[6] Fuhrmann G., Neuer A.L., Herrmann I.K. (2017). Extracellular vesicles—A promising avenue for the detection and treatment of infectious diseases?. Eur. J. Pharm. Biopharm..

[7] Jones L.B., Bell C.R., Bibb K.E., Gu L.-L., Matthews Q.L. (2018). Pathogens and Their Effect on Exosome Biogenesis and Composition. Biomedicines.

[8] Barile L., Vassalli G. (2017). Exosomes: Therapy delivery tools and biomarkers of diseases. Pharmacol. Ther..

[9] Crenshaw Brennetta J., Matthews Qiana S.B. (2018). Biological Function of Exosomes as Diagnostic Markers and Therapeutic Delivery Vehicles in Carcinogenesis and Infectious Diseases. Nanomedicine.

[10] Bell C.R., Jones L.B., Crenshaw B.J., Kumar S., Rowe G.C., Sims B., Javan G.T., Matthews Q.L. (2019). The Role of Lipopolysaccharide-Induced Extracellular Vesicles in Cardiac Cell Death. Biology (Basel).

[11] Jones L.B., Kumar S., Curry A.J., Price J.S., Krendelchtchikov A., Crenshaw B.J., Bell C.R., Williams S.D., Tolliver T.A., Saldanha S.N. (2019). Alcohol Exposure Impacts the Composition of HeLa-Derived Extracellular Vesicles. Biomedicines.

[12] Glebov K., Lochner M., Jabs R., Lau T., Merkel O., Schloss P., Steinhauser C., Walter J. (2015). Serotonin stimulates secretion of exosomes from microglia cells. Glia.

[13] Fitzgerald W., Freeman M.L., Lederman M.M., Vasilieva E., Romero R., Margolis L. (2018). A System of Cytokines Encapsulated in ExtraCellular Vesicles. Sci. Rep..

[14] Jones L.B. (2020). The Effects of Gram Negative Bacteria on Extracellular Vesicles. Doctoral Dissertation.

[15] Holloway B.W., Krishnapillai V., Morgan A.F. (1979). Chromosomal genetics of Pseudomonas. Microbiol. Rev..

[16] Napoli I., Kierdorf K., Neumann H. (2009). Microglial precursors derived from mouse embryonic stem cells. Glia.

[17] Sapan C.V., Lundblad R.L., Price N.C. (1999). Colorimetric protein assay techniques. Biotechnol. Appl. Biochem..

[18] Sims B., Farrow A.L., Williams S.D., Bansal A., Krendelchtchikov A., Gu L.-L., Matthews Q.L. (2017). Role of TIM-4 in exosome-dependent entry of HIV-1 into human immune cells. Int. J. Nanomed..

[19] Wada S., Neinast M., Jang C., Ibrahim Y.H., Lee G., Babu A., Li J., Hoshino A., Rowe G.C., Rhee J. (2016). The tumor suppressor FLCN mediates an alternate mTOR pathway to regulate browning of adipose tissue. Genes Dev..

[20] Crenshaw B.J., Kumar S., Bell C.R., Jones L.B., Williams S.D., Saldanha S.N., Joshi S., Sahu R., Sims B., Matthews Q.L. (2019). Alcohol Modulates the Biogenesis and Composition of Microglia-Derived Exosomes. Biology (Basel).

[21] Wang J., Hendrix A., Hernot S., Lemaire M., De Bruyne E., Van Valckenborgh E., Lahoutte T., De Wever O., Vanderkerken K., Menu E. (2014). Bone marrow stromal cell-derived exosomes as communicators in drug resistance in multiple myeloma cells. Blood.

[22] Cheng X.-T., Xie Y.-X., Zhou B., Huang N., Farfel-Becker T., Sheng Z.-H. (2018). Characterization of LAMP1-labeled nondegradative lysosomal and endocytic compartments in neurons. J. Cell Biol..

[23] Fitzner D., Schnaars M., van Rossum D., Krishnamoorthym G., Dibaj P., Bakhti M., Regen T., Hanisch U.-K., Simons M. (2011). Selective transfer of exosomes from oligodendrocytes to microglia by macropinocytosis. J. Cell Sci..

[24] Rawal S., Manning P., Katare R. (2014). Cardiovascular microRNAs: As modulators and diagnostic biomarkers of diabetic heart disease. Cardiovasc. Diabetol..

[25] Blahna M.T., Hata A. (2013). Regulation of miRNA biogenesis as an integrated component of growth factor signaling. Curr. Opin. Cell Biol..

[26] Calderwood S.K., Mambula S.S., Gray P.J., Theriault J.R. (2007). Extracellular heat shock proteins in cell signaling. FEBS Lett..

[27] Im K., Baek J., Kwon S.Y., Rha S.Y., Hwang K.W., Kim U., Min H. (2018). The Comparison of Exosome and Exosomal Cytokines between Young and Old Individuals with or without Gastric Cancer. Int. J. Gerontol..

[28] Rossi J.-F., Lu Z.-Y., Jourdan M., Klein B. (2015). Interleukin-6 as a Therapeutic Target. Clin. Cancer Res..

[29] Erta M., Quintana A., Hidalgo J. (2012). Interleukin-6, a major cytokine in the central nervous system. Int. J. Biol. Sci..

[30] Yasukawa K., Hirano T., Watanabe Y., Muratani K., Matsuda T., Nakai S., Kishimoto T. (1987). Structure and expression of human B cell stimulatory factor-2 (BSF-2/IL-6) gene. Embo. J..

[31] Chen A.C.-H., Xi Y., Carroll M., Petsky H.L., Gardiner S.J., Pizzutto S.J., Yerkovich S.T., Baines K.J., Gibson P.G., Hodge S. (2017). Cytokine responses to two common respiratory pathogens in children are dependent on interleukin-1beta. ERJ Open Res..

[32] Fulda S., Gorman A.M., Hori O., Samali A. (2010). Cellular stress responses: Cell survival and cell death. Int. J. Cell Biol..

[33] Wall D.M., McCormick B.A. (2014). Bacterial secreted effectors and caspase-3 interactions. Cell. Microbiol..

[34] Chiusolo V., Jacquemin G., Bassoy E.Y., Vinet L., Liguori L., Walch M., Kozjak-Pavlovic V., Martinvalet D. (2017). Granzyme B enters the mitochondria in a Sam50-, Tim22- and mtHsp70-dependent manner to induce apoptosis. Cell Death Differ..

[35] Jiang J.-H., Tong J., Tan K.S., Gabriel K. (2012). From evolution to pathogenesis: The link between β-barrel assembly machineries in the outer membrane of mitochondria and gram-negative bacteria. Int. J. Mol. Sci..

[36] Peleg A.Y., Hooper D.C. (2010). Hospital-acquired infections due to gram-negative bacteria. N. Engl. J. Med..

[37] Valentini M., Gonzalez D., Mavridou D.A., Filloux A. (2018). Lifestyle transitions and adaptive pathogenesis of Pseudomonas aeruginosa. Curr. Opin. Microbiol..

[38] Bhatnagar S., Shinagawa K., Castellino F.J., Schorey J.S. (2007). Exosomes released from macrophages infected with intracellular pathogens stimulate a proinflammatory response in vitro and in vivo. Blood.

[39] Mariani M.M. (2009). Kielian, T. Microglia in infectious diseases of the central nervous system. J. Neuroimmune Pharmacol. Off. J. Soc. NeuroImmune Pharmacol..

[40] Diesselberg C., Ribes S., Seele J., Kaufmann A., Redlich S., Hanisch U.K., Michel U., Nau R., Achutze S. (2018). Activin A increases phagocytosis of Escherichia coli K1 by primary murine microglial cells activated by toll-like receptor agonists. J. Neuroinflammation.

[41] Hoogland I.C.M., Westhoff D., Engelen-Lee J.-Y., Melief J., Seron M.V., Houben-Weerts J.H.M.P., Huitinga I., van Westerloo D.J., van der Poll T., van Gool W.A. (2018). Microglial Activation After Systemic Stimulation With Lipopolysaccharide and Escherichia coli. Front. Cell. Neurosci..

[42] Mayer A.M.S., Hall M.L., Holland M., Castro C., MOlinaro A., Aldulescu M., Frenkel J., Ottenhoff L., Rowley D., Powell J. (2014). Vibrio vulnificus MO6-24/O lipopolysaccharide stimulates superoxide anion, thromboxane B₂, matrix metalloproteinase-9, cytokine and chemokine release by rat brain microglia in vitro. Mar. Drugs.

[43] Dando S.J., Mackay-Sim A., Norton R., Currie B.J., John J.A.S., Ekberg J.A.K., Batzloff M., Ulett G.C., Beacham I.R. (2014). Pathogens penetrating the central nervous system: Infection pathways and the cellular and molecular mechanisms of invasion. Clin. Microbiol. Rev..

[44] Rajendran L., Honsho M., Zahn T.R., Keller P., Geiger K.D., Verkade P., Simons K. (2006). Alzheimer’s disease beta-amyloid peptides are released in association with exosomes. Proc. Natl. Acad. Sci. USA.

[45] Gomes C., Keller S., Altevogt P., Costa J. (2007). Evidence for secretion of Cu,Zn superoxide dismutase via exosomes from a cell model of amyotrophic lateral sclerosis. Neurosci. Lett..

[46] Emmanouilidou E., Melachroinou K., Roumeliotis T., Garbis S.D., Ntzouni M., Margaritis L.H., Stefanis L., Vekrellis K. (2010). Cell-produced alpha-synuclein is secreted in a calcium-dependent manner by exosomes and impacts neuronal survival. J. Neurosci..

[47] Raposo G., Stoorvogel W. (2013). Extracellular vesicles: Exosomes, microvesicles, and friends. J. Cell Biol..

[48] Zhang W.-C., Jiang X.-F., Bao J.-H., Wang Y., Liu H.-X., Tang L.-J. (2018). Exosomes in Pathogen Infections: A Bridge to Deliver Molecules and Link Functions. Front. Immunol..

[49] Sun G., Liu F., Lin T.J. (2005). Identification of Pseudomonas aeruginosa-induced genes in human mast cells using suppression subtractive hybridization: Up-regulation of IL-8 and CCL4 production. Clin. Exp. Immunol..

[50] Gupta A., Pulliam L. (2014). Exosomes as mediators of neuroinflammation. J. Neuroinflammation.

[51] Kim J.Y., Han Y., Lee J.E., Yenari M.A. (2018). The 70-kDa heat shock protein (Hsp70) as a therapeutic target for stroke. Expert Opin. Ther. Targets.

[52] Xi Y., Shao F., Bai X.-Y., Cai G.-Y., Lv Y., Chen X.-M. (2014). Changes in the expression of the Toll-like receptor system in the aging rat kidneys. PLoS ONE.

[53] Kacimi R., Yenari M.A. (2015). Pharmacologic heat shock protein 70 induction confers cytoprotection against inflammation in gliovascular cells. Glia.

[54] Henderson B., Allan E., Coates A.R.M. (2006). Stress wars: The direct role of host and bacterial molecular chaperones in bacterial infection. Infect. Immun..

[55] Neckers L., Tatu U. (2008). Molecular chaperones in pathogen virulence: Emerging new targets for therapy. Cell Host Microbe.

[56] Evans C.G., Chang L., Gestwicki J.E. (2010). Heat shock protein 70 (hsp70) as an emerging drug target. J. Med. Chem..

[57] Muñoz-Carrillo J.L., Contreras-Cordero J.F., Oscar G.-C., Paola T.V.-G., Luis G.R.-G., Viridiana E.H.-R. (2018). Cytokine Profiling Plays a Crucial Role in Activating Immune System to Clear Infectious Pathogens. Immune Response Activation and Immunomodulation.

[58] Barr S.D., Smiley J.R., Bushman F.D. (2008). The interferon response inhibits HIV particle production by induction of TRIM22. PLoS Pathog.

[59] Barbara J.A., Van ostade X., Lopez A. (1996). Tumour necrosis factor-alpha (TNF-alpha): The good, the bad and potentially very effective. Immunol. Cell Biol..

[60] Balkwill F. (2006). TNF-α in promotion and progression of cancer. Cancer Metastasis Rev..

