# Supplementary figures and images for: Effects of Pseudomonas aeruginosa on Microglial-Derived Extracellular Vesicle Biogenesis and Composition
Source: Pathogens. 2019 Dec 14;8(4):297. doi: 10.3390/pathogens8040297 (PMC6963293; doi:10.3390/pathogens8040297)

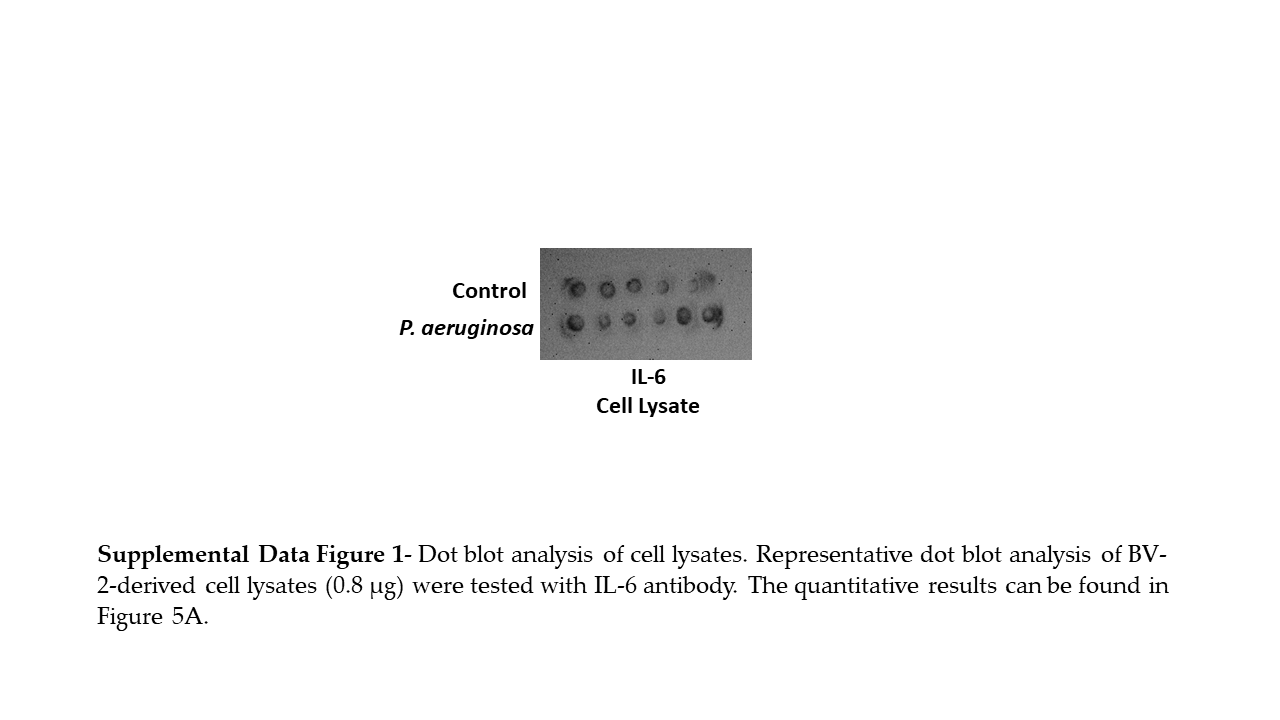

Supplement: Supplementary file 1 [file pathogens-08-00297-s001.zip › Supplimental Figure 1.tif]

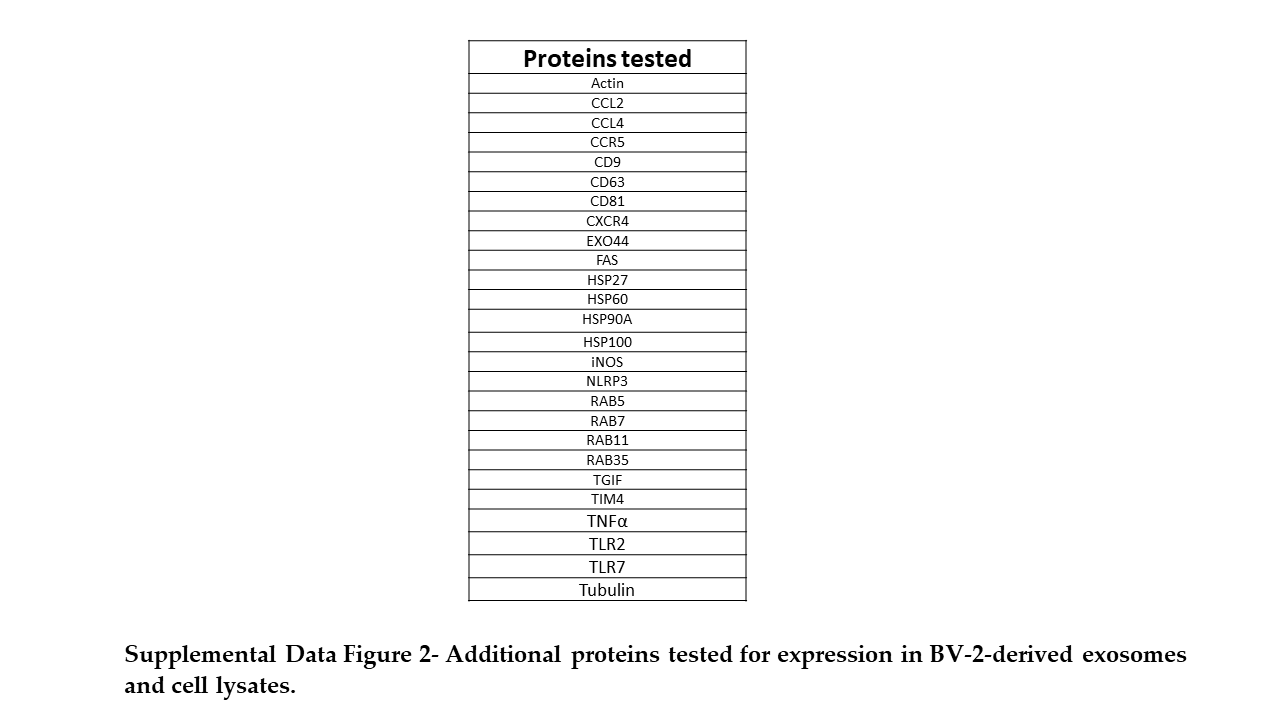

Supplement: Supplementary file 1 [file pathogens-08-00297-s001.zip › Supplimental Figure 2.tif]

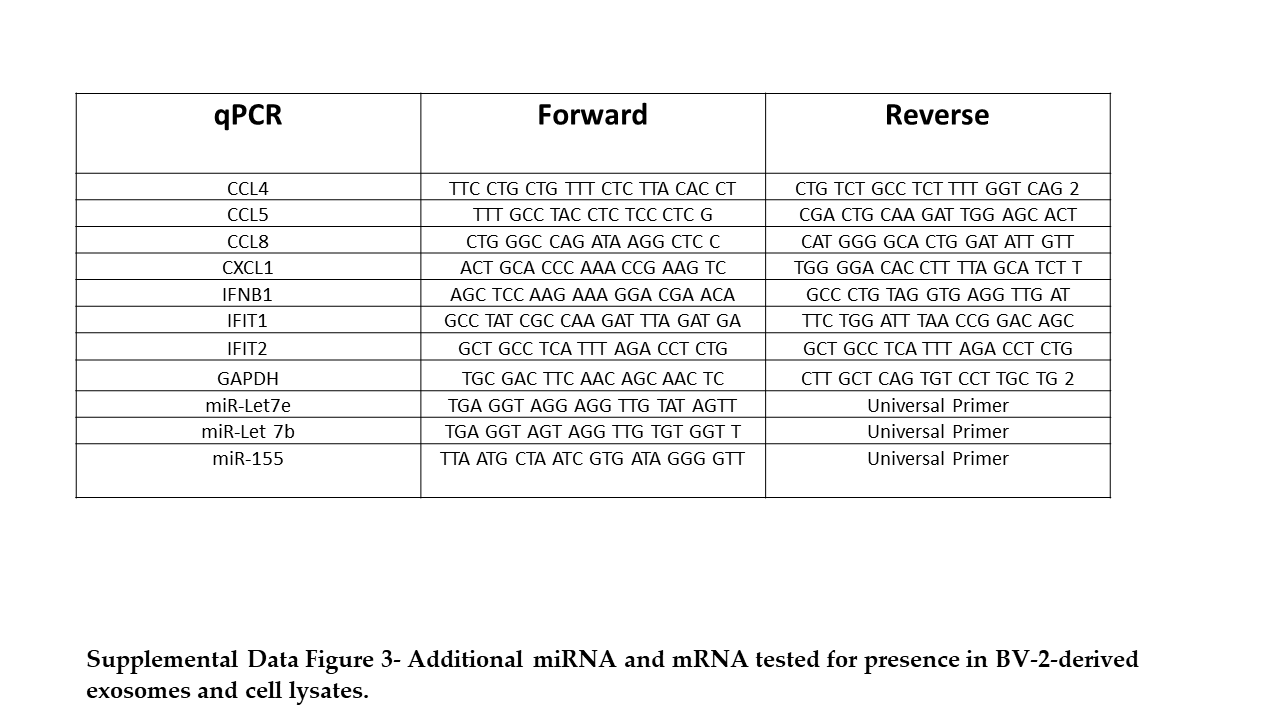

Supplement: Supplementary file 1 [file pathogens-08-00297-s001.zip › Supplimental Figure 3.tif]
